# Outpatient Dental Treatment Expenditure for Patients with Oromaxillofacial Cancer: A Cohort Study in Taiwan

**DOI:** 10.3390/ijerph19031066

**Published:** 2022-01-19

**Authors:** Muhammad Ikbal, Yen-Wen Shen, Wen-Miin Liang, Trong-Neng Wu, Jui-Ting Hsu, Lih-Jyh Fuh

**Affiliations:** 1 School of Dentistry, China Medical University, Taichung 404, Taiwan; ikbal_muhammad@unhas.ac.id (M.I.); a2312830@ms28.hinet.net (Y.-W.S.); 2 Department of Prosthodontic, Faculty of Dentistry, Hasanuddin University, Makassar 90425, Indonesia; 3 Department of Dentistry, China Medical University and Hospital, Taichung 404, Taiwan; 4 Department of Health Services Administration, China Medical University, Taichung 404, Taiwan; wmliang@mail.cmu.edu.tw; 5 Department of Healthcare Administration, Asia University, Taichung 413, Taiwan; tnwu@asia.edu.tw; 6 Department of Bioinformatics and Medical Engineering, Asia University, Taichung 413, Taiwan

**Keywords:** oromaxillofacial cancer, health expenditure, dental treatment, national health programs, cohort study

## Abstract

The information on the outpatient expenditure of patients with oromaxillofacial cancer is minimal. This study aimed to compare the average annual expenditure on dental treatment for these patients 5 years before and 5 years after oromaxillofacial cancer diagnosis. In this study, 7731 patients who received oromaxillofacial cancer diagnosis in 2005 were selected from the Registry of Catastrophic Illness Database as the case-cohort. In the control cohort, 38,655 people without cancer were selected from the National Health Insurance Research Database, with the case–control ratio being 1:5. All participants were observed for 5 years before diagnosis and 5 years after diagnosis. The conditional logistic regression model was used to determine the odds ratios of annual expenditures incurred by participants in the case-cohort. The measurement results indicated that in the oromaxillofacial cancer cohort, the average annual dental expenditure levels at 1, 2, 3, 4, and 5 years after diagnosis were US $97.34, US $77.23, US $109.65, US $128.43, and US $128.03 and those at these years before diagnosis were US $37.52, US $32.10, US $31.86, US $29.14, and US $29.35, respectively. In conclusion, the average annual expenditure on the dental treatment of oromaxillofacial cancer patients after five years of diagnosis was increased compared to five years before diagnosis.

## 1. Introduction

Cancer is a threat to public health and thus warrants considerable attention. Oral cancer accounts for more than half of new cancer cases globally, particularly in Asia [1]. In 2018, oromaxillofacial cancer caused approximately 9.6 million deaths, making it the second dominant cause of death globally. In 2018, approximately 657,000 people received a new diagnosis of oromaxillofacial cancer worldwide [2,3]. Central and South Asia are regions with relatively severe exposure to risk factors for oromaxillofacial cancer. In Taiwan, oromaxillofacial cancer is the fifth most common cause of death in the population and the fourth most common cause of death in men [4,5]. The percentage of people with oromaxillofacial cancer in Taiwan is the highest globally, with 32.46 per 100,000 people. Furthermore, oromaxillofacial cancer has a reasonably high incidence in Taiwan that continues to increase, with the incidence rate being 10.9 times higher in men than in women [5].

Tobacco and alcohol use, chronic inflammation, ultraviolet radiation (for lip cancer), human papillomavirus or candidiasis, immunosuppression, genetic susceptibility, and diet are all risk factors for oral cancer. In addition to dietary deficiencies, tobacco use and alcohol use cause more than 90% of all oral cancers. Oral inflammation was also hypothesized as a risk factor for oral cancer through the involvement of several inflammation-related molecular pathways such as cyclooxygenase-2, epidermal growth factor receptor, p38a mitogen-activated protein kinase, nuclear factor kappa-light-chain-enhancer of activated B cells, and signal transducer and activator of transcription pathways [6]. Moreover, high levels of osteopontin affect the incidence of oral squamous cell carcinoma [7].

Patients with suspected oral cancer should be referred to cancer treatment centers for early detection and treatment by primary care dentists and general practitioners. Improving the expertise of these primary care physicians is critical for increasing the chances of early detection, especially among patients who use tobacco or alcohol in any form. Routine biopsy for patients with precancerous lesions could assist in the early detection of oromaxillofacial cancer. As patients with oromaxillofacial cancer have a high risk of developing malignancies in other head and neck sites as well as in the lungs, upper aerodigestive tract assessment along with history-taking, physical examination, and biopsy must be performed. Screening and prevention may reduce expenditure; moreover, early diagnosis of oromaxillofacial cancer may prevent treatment or overtreatment with high costs. Therefore, comparing expenditure before and after diagnosis is crucial for determining effective preventive and early detection strategies [8,9,10].

Cancer represents a high economic burden for patients, health sectors, and communities [11]. In 2010, the annual economic expenditure for cancer was estimated at US $1.16 trillion based on a total global gross domestic product of >2% [12].

More than 99% of the residents in Taiwan have been covered under the National Health Insurance (NHI) scheme since its implementation in 1995. The government also entrusts Taiwan’s National Health Research Institutes to continually and systemically collect relevant registration and claims data generated in the insurance system. Thus, the collected data are stored in the National Health Insurance Research Database (NHIRD), which contains comprehensive computerized NHI records of the entire population of Taiwan spanning the last 2 decades. We thus consulted this database for the purpose of academic research [13].

Cancer is a cause of significant economic expenditure according to the NHI system in Taiwan. According to the yearly statistical report of the NHI administration, the predicted total medical expenditure for cancer therapy was US $3.1 billion in 2017, and this treatment cost constitutes 38% of the total medical expenditure [2]. Early detection of oromaxillofacial cancer is the most efficient means to reduce medical expenditure, morbidity, disability, and treatment duration and to improve survival, psychological outcomes, and quality of life [1,14,15].

The expenditure associated with oromaxillofacial cancer is enormous, which negatively impacts the health care system and individual families and severely affects the country’s economy. Furthermore, the high expenditure is related to the severity or stage of cancer. The earlier the treatment is conducted, the lower is the expenditure incurred [16,17]. Amarasinghe et al. reported the high expenditure incurred by the government for oromaxillofacial cancer treatment in Sri Lanka; the average annual expenditure for treating a single patient with stage II oromaxillofacial cancer is US $394, and the annual expenditure for treating a patient with stage III or IV oromaxillofacial cancer is US $2027 [18]. Similarly, Yi Huang et al. reported a substantial expenditure of US $28,464–US $81,775 related to cancer treatment in Taiwan [2]. Although considerable information regarding the expenditure for patients with cancer in Taiwan is available, information about the expenditure for patients with oromaxillofacial cancer is minimal. In addition, we hypothesized that the annual average expenditure on dental treatment for patients with oromaxillofacial cancer would increase over a 5-year period from the point of initial diagnosis. Therefore, the purpose of our study was to compare the annual average expenditure on dental treatment for patients between 5 years before and 5 years after diagnosis of oromaxillofacial cancer.

## 2. Materials and Methods

### 2.1. Data Source

The Taiwan Ministry of Health placed all public insurance systems under the NHI program in 1995 to cover the health care of all residents. The National Health Research Institute (NHRI) of Taiwan manages the medical benefit claims of all 22.9 million residents of Taiwan, covering > 99% of the population. The NHRI established several claims data files for public use. We used the NHIRD from the institute, including all claims data from 2000 to 2010. The completeness and accuracy of NHIRD are guaranteed by the Department of Health and the NHI Bureau of Taiwan. The insurance authority released the insured medical records as de-identified secondary data to the public for research. We used the Registry of Catastrophic Illness Patient Database, a subpart of the NHIRD, to identify patients with oromaxillofacial cancer. This study was approved by the Institutional Review Board of China Medical University Hospital (No. CMUH-REC3-074).

### 2.2. Case Definition of Oromaxillofacial Cancer

We selected patients diagnosed with oromaxillofacial cancer for the first time between 1 January and 31 December 2005 from the Registry of Catastrophic Illness Database and added them to the case-cohort. The first day of diagnosis was defined as the index day. Control participants without cancer were selected from the NHIRD. Cases were then matched with controls in a 1:5 ratio [19] according to age, sex, and index day. All participants were observed for 5 years before diagnosis and 5 years after diagnosis for long-term comparison. The annual expenditure incurred due to dental surgery was calculated for all participants during the observation period. Annual expenditure on dental surgery incurred by participants in the case-cohort after cancer diagnosis was compared with that incurred before diagnosis. Furthermore, the expenditure incurred by patients in the case-cohort was matched with that incurred by controls. All cases were observed until 31 December 2010, or until exclusion from the NHI program. Enrolled participants with any history of malignancy before the index day were excluded. The identities of selected participants were concealed at the end of the 5-year follow-up period.

### 2.3. Definition of Outcome

The primary outcome was defined as the dental treatment expenditure of patients diagnosed with oromaxillofacial cancer. Dental treatment expenditure was by calculating the costs of operative dentistry, endodontics, periodontics, and oral surgery in the treatment of patients with oromaxillofacial cancer. The dental treatment expenditure of the patients was calculated over a period of 5 years before and after their diagnosis. The urban level was defined based on living area into five levels of urbanization modified from the classification scheme proposed by Liu et al. [20], who considered the following indicators in determining levels of urbanization: population density, the proportion of residents with a college education or higher, percentage of older adult (>65 years) people, the proportion of the agricultural workforce. In Liu’s study, they divided the urban level into seven levels. The last three levels were combined in this study due to the small number of people.

### 2.4. Statistical Analysis

Differences among groups were evaluated using the *t*-test for continuous variables and the chi-square test for categorical variables. The conditional logistic regression model was used to determine the odds ratios (ORs) of annual expenditures incurred by participants in the case-cohort, which were used to compare the expenditure on dental surgery before and after diagnosis. All surgeries were grouped into one of four types, and separate ORs were calculated for each surgical type. The ORs of annual expenditures incurred by participants in the control cohort for 5 years after the index day compared with the expenditure incurred by the case-cohort during the same period were evaluated. All data management and OR calculations were performed using the SAS software (version 9.3; SAS Institute, Cary, NC, USA).

## 3. Results

The main characteristics of 7731 patients with oromaxillofacial cancer and 38,655 controls are presented in Table 1. The mean age of all patients was 54.92 years, with 88.67% of the patients being men and 11.33% women. In both the case and control cohorts, most of the participants were 40–59 years old (55.97%) and men (88.67%).

In terms of the urban level of residence, the number of patients decreased from the highest to the lowest urban level in both cohorts; the number of patients in the case-cohort decreased from 2566 patients to 1098 patients, and that in the control cohort decreased from 14,157 patients to 4204 patients. Regarding income, the number of patients with oromaxillofacial cancer with incomes <US $360 to incomes >US $1800 tended to decrease. The income of most patients with oromaxillofacial cancer was between US $360 and US $720, accounting for 45.65% of the total.

The 5-year annual average expenditure after the index day was higher than that before the index day in patients with oromaxillofacial cancer (Table 2). In the case-cohort, the annual average dental expenditures for the oromaxillofacial cancer cohort at 1 year, 2 years, 3 years, 4 years, and 5 years were US $97.34, US $77.23, US $109.65, US $128.43, and US $128.03 after the index day and US $37.52, US $32.10, US $31.86, US $29.14, and US $29.35 before the index day, respectively. Nevertheless, the 5-year annual average expenditure after the index day in the case-cohort was higher than that in the control cohort.

**Figure 1:**
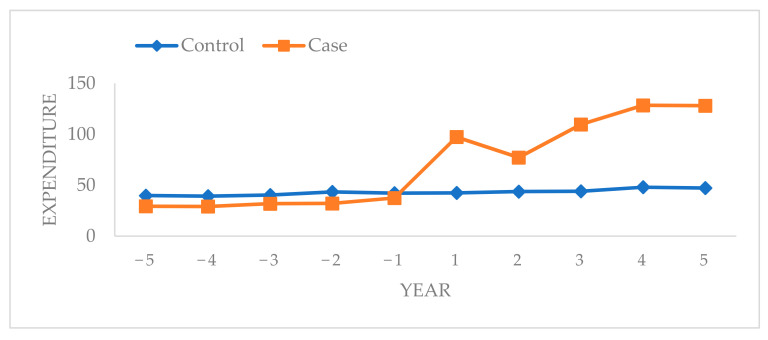
Annual average expenditure before and after the index date in the case and control cohorts.

The graph in Figure 1 shows that the annual average expenditure was lower in the case-cohort than in the control cohort for 5 years before oromaxillofacial cancer was diagnosed. However, the annual average expenditure was higher in the case cohort than in the control cohort 5 years after oromaxillofacial cancer was diagnosed. In addition, the annual average expenditure after diagnosis was higher than that before diagnosis for the patients with oromaxillofacial cancer. The annual average expenditure on patients with oromaxillofacial cancer increased 1 year before diagnosis compared with previous years (Figure 1).

Outpatient oral surgery had the highest annual average expenditure among periodontics, endodontics, and operative dentistry after 5 years of oromaxillofacial cancer diagnosis (Table 3). Regarding operative dentistry treatment, the 4th year before diagnosis had the lowest average expenditure among the 5 years (US $13.77); conversely, the 4th year after diagnosis had the highest mean expenditure (US $40.25). For endodontic treatment, the average expenditure decreased from 1 year to 5 years before diagnosis and fluctuated for 5 years after the diagnosis. Furthermore, the average periodontics treatment expenditure fluctuated from 1 year to 5 years before and after diagnosis. For oral surgical treatment, the average expenditure fluctuated from 1 year to 5 years before diagnosis and significantly increased in the first year after diagnosis. Generally, patients with oromaxillofacial cancer had more average expenditure after the diagnosis than before the diagnosis.

In terms of gender, male patients’ average annual expenditure was highest in the first year of the period before diagnosis and lowest in the fourth year of the period before diagnosis; moreover, their average annual expenditure was highest in the fourth year of the period after diagnosis and lowest in the second year of the period after diagnosis. Female patients’ average annual expenditure was highest in the fourth year of the period before diagnosis and lowest in the fifth year of the period before diagnosis; their average annual expenditure was highest in the fifth year of the period after diagnosis and lowest in the second year of the period after diagnosis. Overall, for both men and women with oromaxillofacial cancer, the average annual expenditure after diagnosis was higher than that before diagnosis.

In terms of age, patients aged ≥ 80 years had the highest average annual expenditure (recorded in the fifth year) during the 5-year period before diagnosis among all age groups, whereas patients aged 0–19 years had the lowest average annual expenditure (recorded in the third year) during the 5-year period before diagnosis among all age groups. During the 5-year period after diagnosis, patients aged 0–19 years had the highest average annual expenditure (recorded in the third year) among all age groups, whereas those aged ≥ 80 years had the lowest average annual expenditure (recorded in the fifth year) among all age groups.

For operative dentistry treatment, the highest annual average expenditure before the diagnosis of oromaxillofacial cancer was at 1 year in patients aged 0–19 years, followed by 2, 3, 4, and 5 years in those aged > 80 years, whereas the highest annual average expenditure after the diagnosis was at 1 year in patients aged 20–39 years, followed by 2, 3, 4, and 5 years in those aged 0–19 years. For endodontics treatment, the highest annual average expenditure before the diagnosis was at 1 year in patients aged 0–19 years, followed by 2 years in patients aged 60–79 years and 3, 4, and 5 years in patients aged >80 years, whereas the highest annual average expenditure after diagnosis was at 1 year in patients aged 60–79 years, followed by 2, 3, 4, and 5 years in patients aged 40–59, 0–19, 40–59, and 0–19 years, respectively. For periodontics treatment, the highest annual average expenditure before the diagnosis was at 1 year and 2 years in patients aged 40–59 years, followed by 3, 4, and 5 years in patients aged 60–79 years, whereas the highest annual average expenditure after the diagnosis was at 1 year in patients aged 40–59 years, followed by 2 years in those aged >80 years and 3, 4, and 5 years in patients aged 40–59 years. For oral surgery treatment, the highest annual average expenditure before the diagnosis was at 1 year, 2 years, 3 years, 4 years, and 5 years in patients aged > 80 years, whereas the highest annual average expenditure after diagnosis was at 1 year in patients aged 20–39 years, followed by 2, 3, 4, and 5 years after diagnosis in patients aged 40–59 years (Table 4).

The conditional logistic regression model revealed that in the first year (OR: 2.23, 95% confidence interval (CI): 2.07–2.40), the annual average expenditures on treatment for patients with oromaxillofacial cancer after diagnosis were two times significantly higher than that before diagnosis (*p* < 0.0001). Furthermore, in the third year (OR: 1.19, 95% Cl: 1.09–1.29), fourth-year (OR: 1.09, 95% Cl: 1.01–1.18), and fifth-year (OR: 1.22, 95% Cl: 1.14–1.32), the annual average expenditure on treatment for patients with oromaxillofacial cancer after diagnosis was higher than that before diagnosis, but the annual average expenditure on treatment was not higher than that in the first year. In the second year (OR: 1.07, 95% CI: 0.99–1.16), the annual average treatment expenditure was higher after diagnosis than before diagnosis but not significant (*p* = 0.0797; Table 5).

## 4. Discussion

Oromaxillofacial cancer has been a significant challenge to public health care in Taiwan. Cancer treatment is closely related to the economic burden on the patient because the treatment expenditure is high. This high medication expenditure includes dental treatment expenditure. Therefore, this study aimed to provide new evidence regarding the average annual expenditures on outpatient dental treatment for patients diagnosed with oromaxillofacial cancer 5 years before and 5 years after the diagnosis. This research was the first study to analyze and explore the average annual expenditure on dental treatment in patients with oromaxillofacial cancer 5 years before and 5 years after the diagnosis in Taiwan. The results revealed that the average annual dental expenditure increased in patients diagnosed with oromaxillofacial cancer in the 5-year follow-up period. This could affect the quality of life of patients and cause difficulty for specific countries or health care systems in paying for the expenditures. The results of this study can serve as a reference for patients and dentists in determining dental treatment expenditure related to oromaxillofacial cancer.

Screening and prevention might reduce the expenditure; if oromaxillofacial cancer is diagnosed earlier, the high cost of treatment or overtreatment may be prevented. Therefore, comparing expenditure before and after the patient received the diagnosis is crucial to determine strategies for prevention or early detection, focusing on risk factors and detection, to reverse the current status—advanced disease as the most frequent diagnosis [9,10].

The study results show that 88.67% of male patients diagnosed with oromaxillofacial cancer, and most patients were in the age group of 40–59 years. The results are in line with those of Conway et al., who stated that the largest percentage of patients with a diagnosis of oromaxillofacial cancer are in Asia, particularly in South-Central Asia, at 48.7%, and the incidence is consistently higher in men than in women, with a male:female ratio of 2:1 [21]. Globally, although oromaxillofacial cancer is commonly diagnosed in men, it can be as prevalent as or even more common in women in several Southeast Asian groups [22]. However, according to Moore et al., the incidence rate for oromaxillofacial cancer among white men in Hawaii has been continuously high for numerous decades [23]. This is probably caused by lifestyle and habit factors; as stated by Gustavo et al., approximately 75% of all oromaxillofacial cancers are attributable to smoking and drinking [24]. Furthermore, Han et al. reported that compared with women, men are more affected by the disease because of their greater exposure to the carcinogenic risk factors linked to oromaxillofacial cancer, such as tobacco and alcohol [25]. Although the costs of diagnosis and hospitalization were similar for both sexes, the treatment costs for men were significantly higher than for women.

This study’s average age group was 40–59 years, and the majority of patients with oromaxillofacial cancer belonged to this age group. This aligns with the results of Han et al. that sex, smoking habit, and age > 40 years are epidemiological risk factors for oromaxillofacial cancer [1,25]. This conclusion, which is in line with prior research, suggests that practitioners focus on patients > 40 years old and encourage them to have a regular oral examination. Regular oral examinations can aid in the early detection of oromaxillofacial cancer. In addition, early treatment of oromaxillofacial cancer can increase patients’ chances of survival while also lowering their medical costs.

The urban level was defined based on living area into five levels of urbanization modified from the classification scheme proposed by Liu et al. [20]. In terms of the urban level of patients’ residence, the number of patients showed a decreasing trend from the highest level to the lowest level in both cohorts, and the lowest urban level had the least number of patients. The medical expenditure burden related to oromaxillofacial cancer treatment was higher for patients living in areas with the lowest urbanization level than for those living in areas with higher urbanization levels. Moreover, the number of patients living in areas with the lowest urbanization level may be limited because of insufficient funds required for the treatment. Expenditure reduction strategies in oromaxillofacial cancer principally should aim to decrease the need for complex reconstructive surgeries and procedures.

Regarding the income, the number of patients with oromaxillofacial cancer tended to decrease from those with low income < NS $360 to high income > US $1800, with 45.65% patients in the income range of US $360–US $720. The reason could be that patients with low income might pay less attention to oral hygiene and hinder the maintenance of good oral hygiene. This is one of the risk factors for oromaxillofacial cancer. This finding is in conjunction with the study conducted by Anwar et al. that men belonging to a low socioeconomic status in their forties who had chewing habits for years constituted the most incidence of oromaxillofacial cancer [26]. The economic burden on patients is a key side effect of cancer therapy, presenting in various forms [2]. Loss of productivity, out-of-pocket spending, bankruptcy, and medical debt are all examples of material problems. Furthermore, financial stress has been related to poor health outcomes in several studies [27]. The economic factors influencing high patient medical expenditures include the lack of medicare, smoking habit, and late clinical-stage [25]. Moreover, as stated by Hemantha et al., the economic burden has been observed in Sri Lanka, negatively affecting both the health care system and individual families, thus seriously affecting the country’s economy [18,28].

Treatment expenses are rapidly increasing due to the increased morbidity and mortality related to oromaxillofacial cancer. In addition to direct costs, such as diagnosis, treatment, and hospitalization, indirect costs are involved, such as patient productivity loss due to illness and impairment, which are difficult to define and calculate [25]. Therefore, in this study, we used data from the NHIRD, which includes all claims data from 2000 to 2010. The accuracy of the NHIRD are guaranteed by the Ministry of Health and Welfare and the NHI Bureau of Taiwan to be major evidence to indicate the expenditure of patients with oromaxillofacial cancer.

This study demonstrated that the annual average expenditure of the case-cohort was lower than that of the control cohort for 5 years before oromaxillofacial cancer was diagnosed. However, the annual average expenditure in the case-cohort was higher than that of the control cohort for 5 years after oromaxillofacial cancer was diagnosed in the 5 years follow-up. In the case-cohort, an increase was observed in the annual average expenditure of dental treatment 1 year before oromaxillofacial cancer diagnosis compared with previous years. Many factors contributed to this result. Depending on the cancer stage and the patient’s condition, oromaxillofacial cancer management often requires a combination of therapies such as multimodal treatment incorporating radiation and chemotherapy. The development of new diagnostic, pharmacological, and treatment technologies and the long-term care of cancer patients contribute to an increased dental treatment expenditure [11]. In addition, late treatment results in the high cost of oromaxillofacial cancer treatment in Taiwan. Chou et al. reported that 67.2% of the 16,691 oromaxillofacial cancer patients were in stage III or IV of the disease when they came for treatment [29].

Oral surgery treatment has the highest annual average expenditure among periodontics, endodontics, and operative dentistry treatment. This is probably because oral surgery is one of the primary treatments of oromaxillofacial cancer, as stated by Han et al. The primary treatments of oromaxillofacial cancer include surgery, radiation, and chemotherapy. These treatments can be used alone or in combination depending on the clinical stage and histology of oromaxillofacial cancer. In addition to primary treatments, patients with oromaxillofacial cancer may require extra care and prior treatments to reduce treatment side effects, such as oral pain from the tumor or oral mucositis, weight loss, fatigue, nausea, vomiting, and altered salivary gland function. At all stages, multimodal treatment consistently resulted in higher expenditures than monotherapies [11,25]. Moreover, Meropol et al. reported that the use of cancer therapeutics is dominated by supportive therapy agents (i.e., hematopoietic growth factors and antiemetics), and anticancer medication is the most expensive among medications administered in outpatient clinics [30].

For the patients with oromaxillofacial cancer, the average annual expenditure on treatment after diagnosis was higher than that before diagnosis. This result is in line with the result reported by Raman et al. in Malaysia that more surgical intervention increased expenditures in the late stages of oromaxillofacial cancer. Patients diagnosed with cancer require additional treatment, such as extensive excisions combined with complex reconstruction procedures. For instance, in the final stages of the surgery, 50% of the methods included resource-intensive microvascular free flap reconstructions, which are expensive. Conversely, some patients in stages I and II required primary excisions and general reconstructive procedures, which are relatively less costly [31]. Treating stage III and IV malignancies were more expensive than treating early-stage tumors, resulting in higher overall spending across the management period [11]. Detection of oromaxillofacial cancer early, cancer prevention, screening of high-risk patients, and early treatment are the most effective methods to reduce costs, limit treatment duration, and improve survival [1,31].

Preventive periodontal and dental therapies are crucial aspects of cancer treatment. Performing timely diagnosis, administering appropriate therapies, and establishing regular maintenance protocols are essential for treating periodontal disease through neutropenic phases and preventing excessive oral bacterial development resulting from radiation-related changes in the head and neck. The critical nature of these therapies is supported by the finding of this study that the highest average annual expenditure for periodontal treatment occurred 1 year after diagnosis for patients aged 40–59 years and 2 years after diagnosis for those aged > 80 years [11,32].

This study had some limitations. First, the study’s participants were all Taiwanese. Therefore, the current findings may not apply to people of different races or countries. Second, the cancer stage was not included in the registration system in this study. Third, this study calculated the direct expenditure on dental treatment for outpatients only. The total expenditures of the patient were not calculated, which should be higher than reported because oromaxillofacial cancer therapy is associated with various indirect costs. Therefore, other costs such as inpatient care and recovery while in hospital should be included in future studies.

## 5. Conclusions

Based on the results of this study, the following conclusions were drawn:

1. The average annual expenditure was higher for male and female patients with oromaxillofacial cancer after diagnosis than before diagnosis.
2. Among the patients diagnosed as having oromaxillofacial cancer, 88.67% were male, and most were aged 40–59 years with low income.
3. The average annual expenditure began to increase 1 year before oromaxillofacial cancer diagnosis in the case group and was two times higher in the first year after diagnosis compared with that before diagnosis.

## Figures and Tables

**Table 1:** Baseline characteristics of the oromaxillofacial cancer case and control cohorts (*n* = 7731; Unit: US $).

	Controls *n* = 38,655	Cases *n* = 7731
Age	54.92 ± 13.21	54.92 ± 13.21
0–19	110 (0.28)	22 (0.28)
20–39	4240 (10.97)	848 (10.97)
40–59	21,634 (55.97)	4327 (55.97)
60–79	11,319 (29.28)	2264 (29.28)
≥80	1352 (3.50)	270 (3.49)
Sex		
Male	34,275 (88.67)	6855 (88.67)
Female	4380 (11.33)	876 (11.33)
Urban level		
Highest	12,830 (33.19)	2566 (33.19)
High	9040 (23.39)	1808 (23.39)
Middle	5695 (14.73)	1139 (14.73)
Low	5600 (14.49)	1120 (14.49)
Lowest	5490 (14.20)	1098 (14.20)
Income		
<360	11,990 (31.02)	2398 (31.02)
360–720	17,645 (45.65)	3529 (45.65)
>720–1080	3705 (9.58)	741 (9.58)
>1080–1440	2140 (5.54)	428 (5.54)
>1440–1800	1780 (4.6)	356 (4.60)
>1800	1395 (3.61)	279 (3.61)

**Table 2:** The average expenditure of oromaxillofacial cancer case and control cohorts.

	Controls *n* = 38,655	Cases *n* = 7731
Average expenditure		
Before index day		
5 years	39.81	29.35
4 years	39.27	29.14
3 years	40.41	31.86
2 years	43.47	32.10
1 year	42.24	37.52
After index day		
1 year	42.46	97.34
2 years	43.76	77.23
3 years	44.06	109.65
4 years	48.08	128.43
5 years	47.22	128.03

**Table 3:** Annual average expenditure of patients diagnosed with oromaxillofacial cancer (*n* = 7731; Unit: NT$; US $1 = NT $27.82).

	Before					After				
	5 Years	4 Years	3 Years	2 Years	1 Year	1 Year	2 Years	3 Years	4 Years	5 Years
Age (years)										
0–19	56.56	49.39	19.21	142.40	156.75	146.61	181.37	285.39	164.29	216.23
20–39	50.93	43.15	49.77	61.16	61.52	234.04	104.04	124.67	129.38	146.66
40–59	50.86	54.54	58.16	57.44	71.67	197.78	126.05	140.68	157.35	140.72
60–79	88.77	86.08	96.82	91.63	102.71	198.34	93.73	121.80	110.36	89.83
≥80	161.48	110.27	123.99	114.60	122.56	99.90	69.90	39.50	22.29	21.57
Sex										
Male	27.86	26.13	30.49	29.59	36.21	96.01	76.58	107.17	127.11	126.36
Female	41.04	52.63	42.55	51.81	47.73	107.70	81.70	125.50	136.42	137.82
Treatment										
Operative dentistry	14.49	13.77	15.96	15.43	16.11	28.24	32.63	38.47	40.25	39.79
Endodontics	9.52	9.41	10.15	10.87	11.26	18.47	23.29	31.72	31.32	30.63
Periodontics	0.40	0.39	0.34	0.39	0.57	1.34	0.87	0.95	0.99	1.14
Oral surgery	4.95	5.56	5.40	5.41	9.58	49.28	20.44	38.51	55.87	56.47

**Table 4:** Annual average expenditure in oromaxillofacial cancer (stratified by treatment; Unit: US $).

Age (Years)	Before					After				
	5 Years	4 Years	3 Years	2 Years	1 Year	1 Year	2 Years	3 Years	4 Years	5 Years
Operative dentistry										
0–19	40.25	31.88	19.21	128.56	105.59	30.19	158.06	158.87	153.95	141.46
20–39	38.20	23.79	28.96	33.12	36.33	77.28	52.30	51.97	51.87	52.61
40–59	24.70	26.72	29.76	28.96	29.22	55.15	52.44	51.00	48.12	43.55
60–79	36.60	36.00	45.15	37.63	40.74	56.33	34.62	32.36	27.98	22.75
≥80	77.89	52.42	49.07	45.51	56.34	49.03	29.52	10.56	10.95	0.60
Endodontics										
0–19	16.31	14.87	0.00	12.60	43.00	0.00	23.31	87.23	10.33	68.53
20–39	8.90	12.90	14.74	22.66	14.43	36.39	20.83	30.26	20.70	32.75
40–59	16.81	17.21	18.69	18.71	22.09	38.55	39.91	39.64	38.55	34.21
60–79	33.07	29.58	31.36	33.11	32.92	40.86	30.89	41.81	34.01	21.14
≥80	47.62	34.89	38.48	30.91	23.21	16.17	18.62	4.92	3.24	5.85
Periodontics										
0–19	0.00	0.00	0.00	0.00	0.00	0.00	0.00	0.00	0.00	0.00
20–39	0.00	0.00	0.00	0.00	0.43	2.61	2.02	0.85	0.29	1.37
40–59	0.98	0.56	0.83	0.98	1.39	3.23	1.00	1.34	1.44	1.41
60–79	1.11	2.01	0.93	0.97	1.00	1.77	1.49	1.00	0.71	0.41
≥80	0.00	0.37	0.00	0.00	4.02	2.32	2.76	0.00	0.74	0.00
Oral surgery										
0–19	0.00	2.64	0.00	1.24	8.15	116.42	0.00	39.29	0.00	6.25
20–39	3.83	6.46	6.07	5.37	10.34	117.75	28.89	41.59	56.52	59.94
40–59	8.37	10.06	8.87	8.79	18.98	100.85	32.70	48.70	69.25	61.56
60–79	17.99	18.48	19.38	19.91	28.05	99.38	26.73	46.63	47.66	45.52
≥80	35.98	22.59	36.44	38.17	38.99	32.39	19.00	24.03	7.35	15.13
	USD $									

**Table 5:** Conditional logistic regression model.

After vs. Before	Adjusted OR (95% CI) *	*p*-Value
1 year	2.23 (2.07–2.40)	<0.0001
2 years	1.07 (0.99–1.16)	0.0797
3 years	1.19 (1.09–1.29)	<0.0001
4 years	1.09 (1.01–1.18)	<0.0001
5 years	1.22 (1.14–1.32)	<0.0001

* Adjusted for age, sex, urban level, and income.

## Data Availability

The data sets used and analyzed during the current study are available from the corresponding author upon reasonable request.
